# Activation of Ustilaginoidin Biosynthesis Gene *uvpks1* in *Villosiclava virens* Albino Strain LN02 Influences Development, Stress Responses, and Inhibition of Rice Seed Germination

**DOI:** 10.3390/jof10010031

**Published:** 2023-12-31

**Authors:** Mengyao Xue, Xuwen Hou, Gan Gu, Jie Dong, Yonglin Yang, Xiaoqian Pan, Xuan Zhang, Dan Xu, Daowan Lai, Ligang Zhou

**Affiliations:** Department of Plant Pathology, College of Plant Protection, China Agricultural University, Beijing 100193, China; mengyaoxue@cau.edu.cn (M.X.); xwhou@cau.edu.cn (X.H.); gangu@cau.edu.cn (G.G.); jiedong@cau.edu.cn (J.D.); yonglinyang@cau.edu.cn (Y.Y.); xiaoqianpan@cau.edu.cn (X.P.); xzhang@cau.edu.cn (X.Z.); cauxudan@cau.edu.cn (D.X.); dwlai@cau.edu.cn (D.L.)

**Keywords:** rice false smut, *Villosiclava virens* (*Ustilaginoidea virens*), white rice false smut balls, biological functions, mycotoxins, phytotoxins, stress tolerance, phenotype, sporulation

## Abstract

*Villosiclava virens* (anamorph: *Ustilaginoidea virens*) is the pathogen of rice false smut (RFS), which is a destructive rice fungal disease. The albino strain LN02 is a natural white-phenotype mutant of *V. virens* due to its incapability to produce toxic ustilaginoidins. In this study, three strains including the normal strain P1, albino strain LN02, and complemented strain *uvpks1*^C^-1 of the LN02 strain were employed to investigate the activation of the ustilaginoidin biosynthesis gene *uvpks1* in the albino strain LN02 to influence sporulation, conidia germination, pigment production, stress responses, and the inhibition of rice seed germination. The activation of the ustilaginoidin biosynthesis gene *uvpks1* increased fungal tolerances to NaCl-induced osmotic stress, Congo-red-induced cell wall stress, SDS-induced cell membrane stress, and H_2_O_2_-induced oxidative stress. The activation of *uvpks1* also increased sporulation, conidia germination, pigment production, and the inhibition of rice seed germination. In addition, the activation of *uvpks1* was able to increase the mycelial growth of the *V. virens* albino strain LN02 at 23 °C and a pH from 5.5 to 7.5. The findings help in understanding the effects of the activation of *uvpks1* in albino strain LN02 on development, pigment production, stress responses, and the inhibition of rice seed germination by controlling ustilaginoidin biosynthesis.

## 1. Introduction

Rice false smut (RFS), caused by *Villosiclava virens* (anamorph: *Ustilaginoidea virens*), is a destructive rice disease in rice-producing areas worldwide. Moreover, the RFS pathogen (*V. virens*) can produce mycotoxins to decrease the yield and quality of grains [1,2,3,4]. White rice false smut was first reported in Japan in 1991 [5]. A symptom of RFS-infested grains is white instead of normal yellowish-green smut balls growing on the panicles. It was later reported in China [6,7] and the United States [8]. Jin et al. described the isolation and biological features of two albinotic isolates of *V. virens*, wherein the RFS balls remained completely white on the infected panicles, and the surfaces of the chlamydospores were less verrucose than those of the normal chlamydospores of *V. virens* [7].

The secondary metabolites are crucial players in fungal development and interactions with other organisms [9,10]. It is speculated that the mycotoxins produced by the RFS pathogen have many physiological and ecological functions such as influences on sporulation, mycelial growth and pathogenesis, and protection against environmental stresses. In our previous study [11], four sorbicillinoids (i.e., trichotetronine, demethyltrichodimerol, dihydrotrichodimer ether A, and bisorbicillinol) as the main mycotoxins were isolated from the albino strain LN02 of *V. virens.* However, the toxic ustilaginoidins were not found due to the four-base deletion in the promoter sequence of the polyketide synthase (PKS)-encoding gene *uvpks1* in strain LN02. The mutation of the *uvpks1* promoter led to the silencing of the ustilaginoidin biosynthetic gene cluster (BGC) and the elimination of colored ustilaginoidin production. The normal *uvpks1* complemented mutant of strain LN02 could restore the expression of the *uvpks1* gene and the ability to synthesize ustilaginoidins. Further investigation showed that *V. virens* was a non-melanin-producing fungus. The deficiency of ustilaginoidin biosynthesis was proven to be the cause of albinism in the albino strain LN02 [11].

In this study, the ustilaginoidin biosynthesis gene *uvpks1* in the albino strain LN02 was activated via complementation to describe its effects on development, pigment production, and resistance to environmental stresses, as well as the inhibition of rice seed germination, in order to reveal the biological functions of the *uvpks1* gene and ustilaginoidins in the RFS pathogen *V. virens*.

## 2. Materials and Methods

### 2.1. Fungal Strains

The albino strain LN02 of *V. virens* was isolated from white RFS balls [11]. The normal strain P1 of *V. virens* was kindly provided by Prof. Wenxian Sun from the Department of Plant Pathology, China Agricultural University. Three complemented strains, *uvpks1^C^*-1, *uvpks1^C^*-2, and *uvpks1^C^*-3, were obtained by cloning the genomic fragments, including the native promoter from strain P1 plus the coding sequence (CDS) of *uvpks1* from strain LN02, into the pCBHT binary vector, which was then introduced into the protoplasts of strain LN02. As all three complemented strains displayed very similar phenotypes, only the *uvpks^C^*-1 strain was selected in this study. The transgenic strains were grown in an incubator at 28 °C. The pCBHT vector was kindly provided by Prof. Jin-Rong Xu from the Department of Botany and Plant Pathology, Purdue University. The detailed methods are described in the Supplementary Materials and our previous study [11]. The primers used in this study are shown in Table S1. The LN02, P1, and *uvpks1^C^*-1 strains, were transferred to potato dextrose broth (PDB) containing 50% glycerol and were stored at −80 °C at China Agricultural University.

### 2.2. Assessment of Sporulation and Conidia Germination

To assess the effects of the activation of *uvpks1* on sporulation and conidia germination, the P1, LN02, and *uvpks1*^C^-1 strains of *V. virens* were cultured in potato sucrose broth (PSB, comprising 200 g/L of potato and 20 g/L of sucrose). The conidia were obtained via the filtration of the culture broths after the fungal strains were cultured in PSB medium at 28 °C for 7 days. The conidia quantity and germination rates were determined with an optical microscope and a hemocytometer according to previously described methods [12,13,14].

### 2.3. Transient Expression Assay

The transient expression assay was performed according to the method previously described in [15]. Briefly, the green fluorescent protein (GFP)-encoding regions under the control of the promoters from the P1 and LN02 strains in the pCBHT-GFP vector were introduced into the leaf cells of *Nicotiana tabacum* with the T-DNA from *Agrobacterium tumefaciens.* The treated tobacco plants were kept in a greenhouse at 25 °C. GFP fluorescent signals were detected using a confocal laser scanning microscope (Zeiss LSM710 3-channel, Zeiss Company, Jena, Germany).

### 2.4. Effects of Environmental Stresses on Mycelial Growth of Fungal Strains

An amount of 1 μL of a spore suspension (1 × 10^6^ spores/mL) of *V. virens* was inoculated in the center of a plate at 28 °C and pH 6.5 for 18 days. Mycelial growth was assayed after incubation at 28 °C and pH 6.5 for 18 days on plates of potato sucrose agar (PSA, containing 200 g/L of potato, 20 g/L of sucrose, and 20 g/L of agar), and the PSA was treated with 0.1–0.4 M of sodium chloride (NaCl); 2 mg/mL or 4 mg/mL of Congo red (CR); 0.025% or 0.050% sodium dodecyl sulfate (SDS, *w*/*v*); and 0.02% or 0.04% hydrogen peroxide (H_2_O_2_, *v*/*v*), respectively. The colony diameters were measured, and the mycelial growth of the treated strains was compared with that of non-treated controls [16]. The inhibition ratio was calculated using the following equation: [(average colony diameter of each strain on PSA without treatment—average colony diameter of each strain on PSA with treatment)/average colony diameter of each strain on PSA without treatment] × 100%. All experiments were repeated three times with three replicates each time.

### 2.5. Effects of Fungal Extracts on Germination of Rice Seeds

The fungal strains P1, LN02, and *uvpks1*^C^-1 were separately grown on PDA medium in Petri dishes at 28 °C for 7 days. Then, three agar plugs (0.5 cm × 0.5 cm) with mycelia were added into a 1000 mL Erlenmeyer flask containing 500 mL of PDB under aseptic conditions and incubated at 28 °C in darkness for 30 days in a rotary shaker at 180 rpm.

The liquid cultures of fungal strains were extracted with ethyl acetate (EtOAc) at room temperature three times. The EtOAc extract was concentrated under vacuum at 42 °C in a rotatory evaporator. The obtained brownish residue was then diluted with dimethylsulfoxide (DMSO) for the assessment of the radicle and plumule elongation inhibitory effects on the seed germination of cultivated rice varieties 9311 and Zhonghua 17. The rice seeds were kindly provided by Prof. Zejian Guo from the Department of Plant Pathology, China Agricultural University. The assay was performed in a 24-well plate using the method described previously in [17]. Briefly, the three-day-germinated rice seeds of each rice variety were sown directly into each well containing 200 μL of a working solution in a 24-well plate. The DMSO solutions containing the EtOAc extracts of the fungal cultures were then added to sterile distilled water with a DMSO concentration of 2.5% and an EtOAc extract concentration of 10 μg/mL. The 2.5% DMSO in distilled water was used as the negative control. Three replicates were used for each treatment. The plates were incubated in a moist chamber at 25 °C in darkness. The length of each radicle or plumule was measured after a treatment period of 48 h. The inhibition rate of radicle or plumule elongation was calculated as follows: inhibition rate = [(Lc − Lt)/Lc] × 100%, where Lc is the radicle or plumule length of the non-treated group, and Lt is that of the treated group.

### 2.6. Statistical Analysis

All experiments were designed with three independent biological replicates. Five replicates were performed for each treatment. The treated samples were analyzed at the same growth stage. Extreme individual samples were excluded. All statistical analyses were conducted using SPSS version 17.0 (SPSS, Inc., Chicago, IL, USA). Comparisons were tested for statistical significance with Student’s *t*-test. The data were expressed as the means ± standard error (SE). Differences at *p* < 0.05 or *p* < 0.01 were considered statistically significant or extremely significant.

## 3. Results

### 3.1. Activation of uvpks1 Increased Sporulation and Conidia Germination

**Figure 1:**
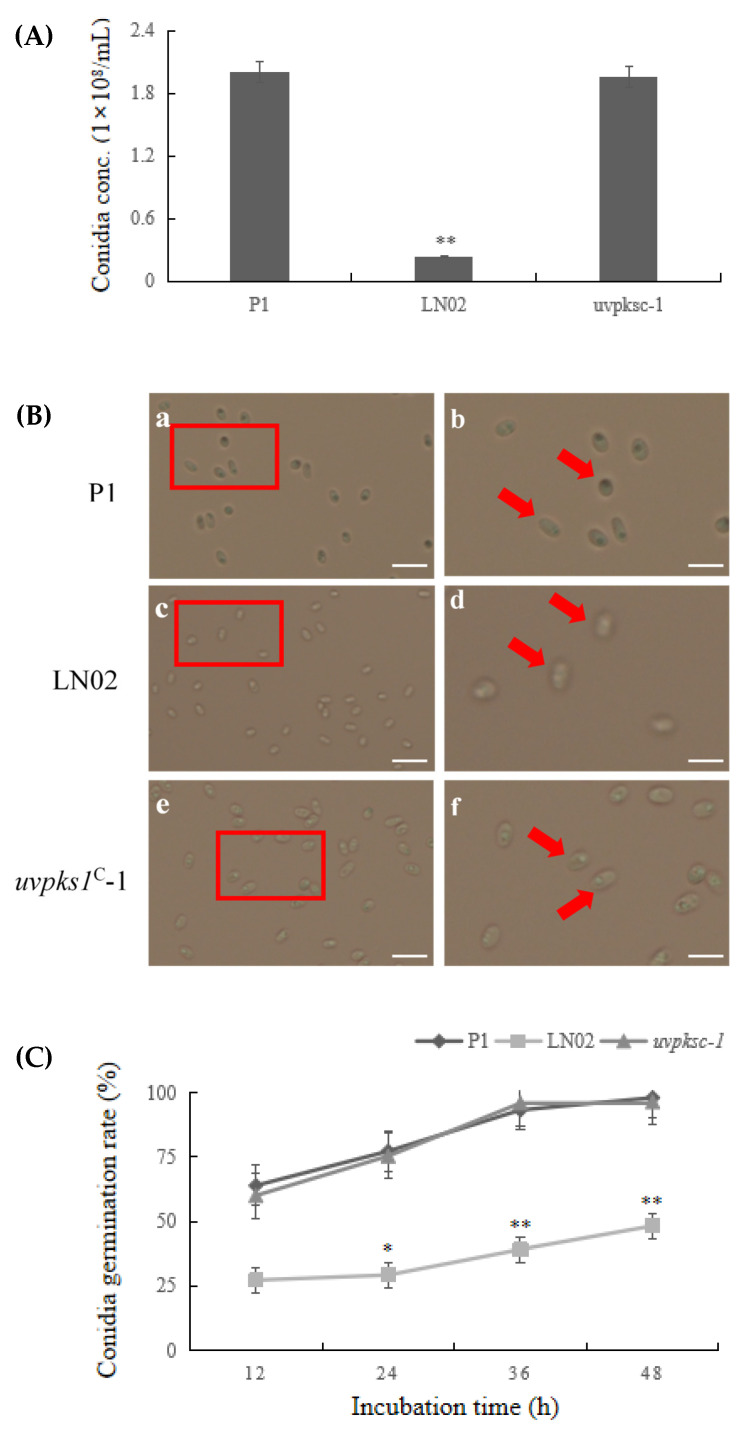
Comparison of the sporulation and conidia germination between fungal strains. (**A**) The conidia concentrations in PSB medium at 28 °C, pH 6.5, and 180 rpm over 7 days. (**B**) The conidia morphology observed under an optical microscope. The conidia were collected via the filtration of the culture broth after the strains were cultured in PSB medium at 28 °C and 180 rpm for 7 days. The photos in (**Bb**,**Bd**,**Bf**) are the enlarged views of the photos of the red squares in (**Ba**,**Bc**,**Be**), respectively. (**C**) The germination rates of conidia collected from PSB medium at 28 °C and 180 rpm after 12, 24, 36, and 48 h, respectively. The data are indicated as means ± SD (from at least three independent samples) and were compared with P1 strain using Student’s *t*-test (* *p* < 0.05; ** *p* < 0.01). Scale bars: 20 μm in (**Ba,Bc**,**Be**); 5 μm in (**Bb**,**Bd**,**Bf**); The red arrowheads indicate the typical spores in (**B**).

Secondary metabolites can influence fungal development, such as mycelial growth, sporulation, conidia germination, and sexual reproduction [18,19]. In order to investigate the functions of *uvpks1*, the P1, LN02, and *uvpks1*^C^-1 strains of *V. virens* were cultured in PSB medium. It was found that the conidia concentration (1.96 × 10^8^ conidia/mL) of the complemented strain *uvpks1*^C^-1 was restored to the same level as that (2.00 × 10^8^ conidia/mL) of the P1 strain after 7 days of fermentation in PSB medium. The conidia concentration (0.23 × 10^8^ conidia/mL) decreased by approximately 10-fold in the LN02 strain compared with the P1 strain or the *uvpks1*^C^- 1 strain (Figure 1A).

The conidia of the three strains were obtained via the filtration of the culture broths after they were cultured in PSB medium for 7 days. The green color of the conidia surface, especially inside the conidia of the P1 strain, was observed obviously. However, the conidia color of the LN02 strain appeared to be white under the optical microscope. It seemed that the light-colored compounds were accumulated inside the conidia of the complemented strain *uvpks1*^C^-1. The red arrowheads indicate the typical spores in Figure 1B. Meanwhile, the conidia germination rates of the three strains were compared in different cultivation periods from 12 h to 48 h. The germination rate of the LN02 strain spores was significantly lower than that of the P1 strain or *uvpks1^C^*-1 strain at each incubation time (Figure 1C). The results indicate that the activation of *uvpks1* could increase the sporulation and conidia germination rate in the *uvpks1*^C^-1 strain.

**Figure 2:**
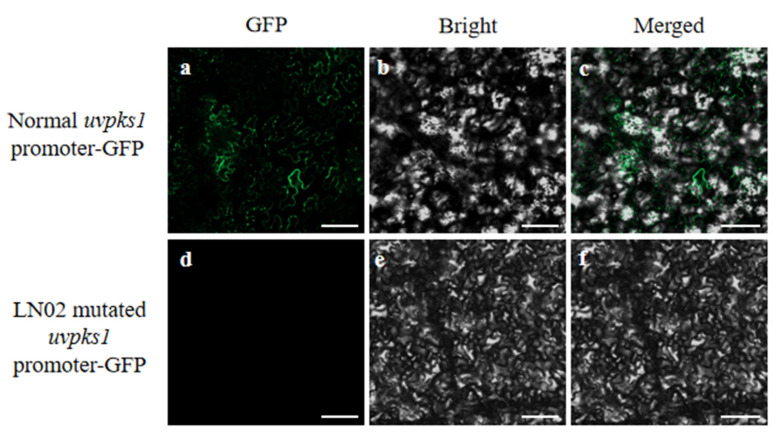
Transient expression of the promoters in the leaf cells of *Nicotiana tabacum*. Transient expression of promoters: normal *uvpks1* promoter–GFP (**a**–**c**) and LN02 mutated *uvpks1* promoter–GFP (**d**–**f**) fusion proteins located in the leaf cells of *N. tabacum*, respectively. Scale bars in the photos: 20 μm.

Furthermore, we performed a transient expression assay on the promoter fragments of *uvpks1* from either the LN02 strain or P1 strain in the leaf cells of *Nicotiana tabacum* to test the effects of the promoter region. A green fluorescent protein (GFP) fusion construct driven by the tested promoter was generated, which contained either a normal native *uvpks1* promoter or a mutated *uvpks1* promoter in front of the GFP-encoding gene. The protein was named as either normal *uvpks1* promoter–GFP or LN02 mutated *uvpks1* promoter–GFP. Compared with the expression activity of the normal *uvpks1* promoter, the relative expression activity of the deletion-mutated promoter in the LN02 strain was not observed. This indicates that the promoter fragment of *uvpks1* from the LN02 strain did not have any activity in the leaf cells of *N. tabacum* (Figure 2), which confirmed that the inactive promoter of *uvpks1* in the LN02 strain led to the silencing of *uvpks1* expression in the albino strain.

### 3.2. Activation of uvpks1 Influenced Fungal Resistance to Environmental Stresses

Stress tolerance is one of the most important mechanisms for the survival of fungi. Environmental stresses, such as temperature, pH, UV, and hyperosmotic stress, have especially critical effects on fungal growth, cellular composition, and metabolism, which have roles in virulence, pathogenicity, adaptation to the environment, colonization, and interactions with host plants and other organisms [20,21,22,23].

#### 3.2.1. Activation of *uvpks1* Increased Mycelial Growth at Relatively low Temperature

**Figure 3:**
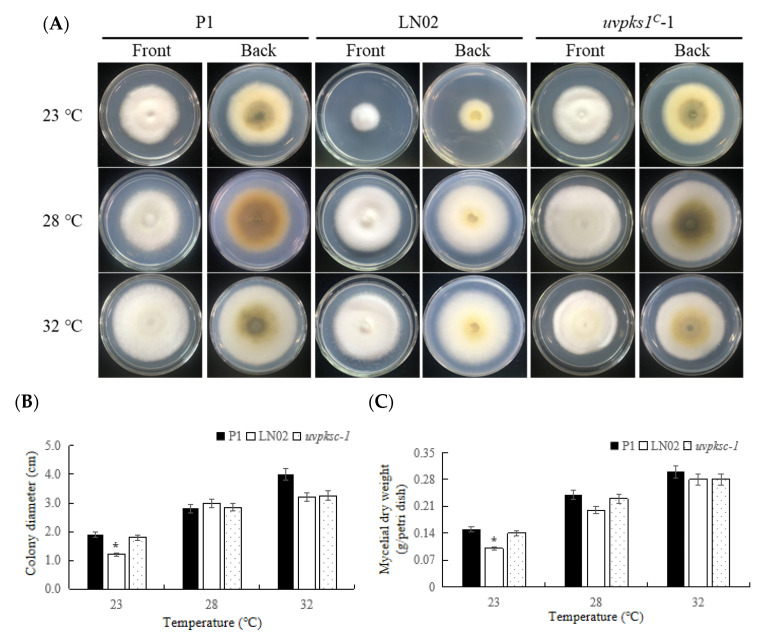
Effects of temperature on growth of fungal strains P1, LN02, and *uvpks1^C^*-1. (**A**) The colonies of fungal strains growing on PSA medium at pH 6.5 for 21 days after inoculation at 23 °C, 28 °C, and 32 °C, respectively. (**B**) The colony expansion diameters of fungal strains. (**C**) The mycelial dry weight per Petri dish. *, *p* < 0.05.

Fungi are able to grow and survive at relatively low temperatures. They usually evolve various strategies such as the production of secondary metabolites in response to low temperatures [24]. When the fungal strains P1, LN02, and *uvpks1^C^*-1 were exposed for 21 days after inoculation to temperatures of 23 °C, 28 °C, and 32 °C, respectively, their growth trends were similar. As the temperature was increased, the fungal growth was accelerated (Figure 3). The LN02 strain produced fewer pigments and grew relatively slowly at 23 °C. However, the *uvpks^C^-*1 strain produced more pigments (Figure 3A) and restored fungal growth based on colony expansion and mycelial dry weight (Figure 3B,C). It was indicated that the normal *uvpks1* in *V. virens* played a role in producing more pigments in response to low-temperature stress (i.e., 23 °C). The pigments were previously identified mainly as bis-naphtho-γ-pyrones, including ustilaginoidins E, K, and D, and isochaetochromin B2 in the *uvpks1^C^*-1 and P1 strains [11].

#### 3.2.2. Activation of *uvpks1* Increased Fungal Growth at pH Values from 5.5 to 7.5

In many fungal pathogens, the PacC transcription factor regulates environmental adaptation, secondary metabolism, and virulence [25]. A low pH often favors the production of mycotoxins, such as deoxynivalenol production by *Fusarium graminearum* [26].

**Figure 4:**
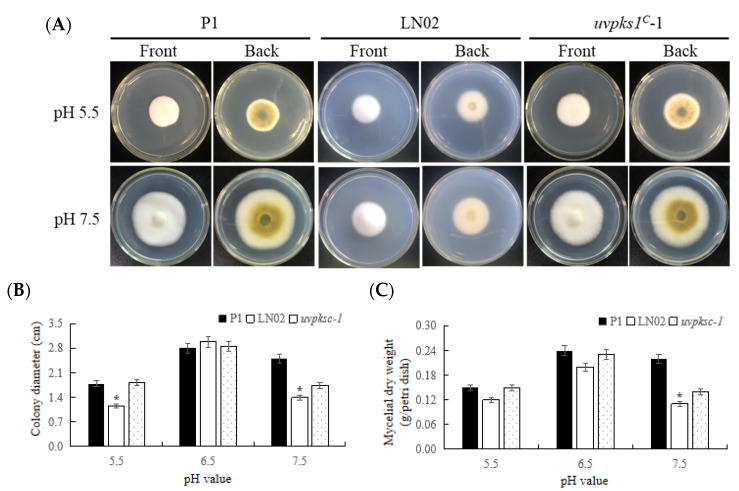
Effects of pH values in medium on growth of fungal strains P1, LN02, and *uvpks1^C^*-1. (**A**) The colonies of fungal strains grew for 21 days after inoculation with pH values of 5.5, 6.5, and 7.5 in PSA medium at 28 °C, respectively. The photos of the three strains at pH 6.5 are the same as those at 28 °C and pH 6.5 in Figure 3A. (**B**) The colony expansion diameters of fungal strains. (**C**) The mycelial dry weight per Petri dish. *, *p* < 0.05.

The PSA medium was treated with Tris-HCl, with its pH values regulated to 5.5, 6.5, and 7.5, respectively. The P1, LN02, and *uvpks1^C^-1* strains were subjected to pH stress. After 21 days of culture, the colony expansion diameters of the fungal strains showed significant differences (Figure 4). The growth of the LN02 strain was more strongly inhibited by pH stress than that of the P1 strain or *uvpks1^C^*-1 strain (Figure 4C). The results show that the activation of *uvpks1* could increase fungal growth and pigment formation at pH values from 5.5 to 7.5 (Figure 4A).

#### 3.2.3. Activation of *uvpks1* Increased Fungal Resistance to NaCl-Induced Osmotic Stress

**Figure 5:**
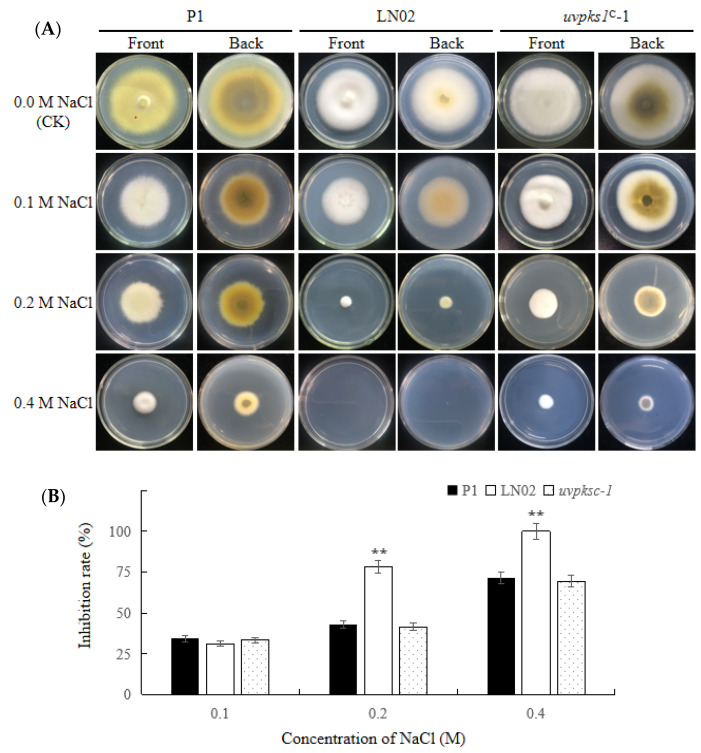
Effects of NaCl in medium on growth of fungal strains P1, LN02, and *uvpks1^C^*-1. (**A**) The colonies of fungal strains grown for 21 days treated with NaCl at concentrations of 0.1, 0.2, and 0.4 M in PSA medium, respectively. (**B**) Inhibition of the colony expansion diameters of fungal strains. **, *p* < 0.01.

The production of secondary metabolites is usually increased in fungi in response to increasing osmotic stress [27]. When the fungal strains P1, LN02, and *uvpks1^C^*-1 were exposed to hyperosmotic stress induced by NaCl at 0.1–0.4 M in PSA medium, the growth rates of all strains were decreased as the concentration of NaCl was increased (Figure 5). However, the LN02 strain displayed more sensitivity and produced fewer pigments under salt stress compared with the P1 and *uvpks1*^C^-1 strains (Figure 5A). These results indicate that the activation of *uvpks1* could increase fungal resistance to osmotic stress induced by NaCl.

#### 3.2.4. Activation of *uvpks1* Increased Fungal Resistances to Congo Red-Induced Cell Wall Stress

**Figure 6:**
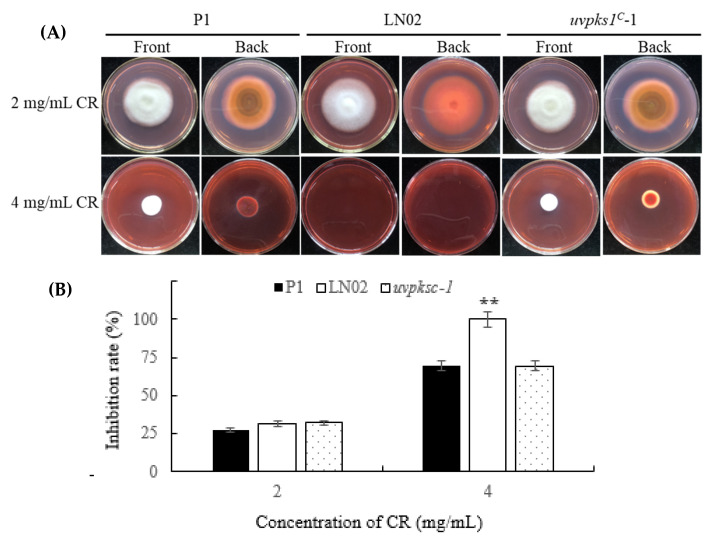
Effects of Congo red in medium on growth of fungal strains P1, LN02, and *uvpks1^C^*-1. (**A**) The colonies of fungal strains grew for 21 days treated with Congo red at concentrations of 2 mg/mL and 4 mg/mL in PSA medium, respectively. The CK (0 mg/mL CR) photos of the three strains are the same as those (0.0 M NaCl) in Figure 5A. (**B**) Inhibition of the colony expansion diameters of fungal strains. **, *p* < 0.01.

Secondary metabolites can increase fungal resistance to cell wall impairment. The production of some secondary metabolites can be induced in response to cell wall stress [28]. The P1, LN02, and *uvpks1^C^*-1 strains were subjected to Congo red (CR)-induced cell wall stress. When the fungal strains were cultured in the medium containing CR, the evidence was that the growth and pigment production of strain LN02 were more strongly inhibited than those of strains P1 and *uvpks1^C^*-1. These results show that the activation of *uvpks1* could increase fungal resistance to cell wall stress induced by CR (Figure 6).

#### 3.2.5. Activation of *uvpks1* Increased Resistance to SDS-Induced Cell Membrane Stress

**Figure 7:**
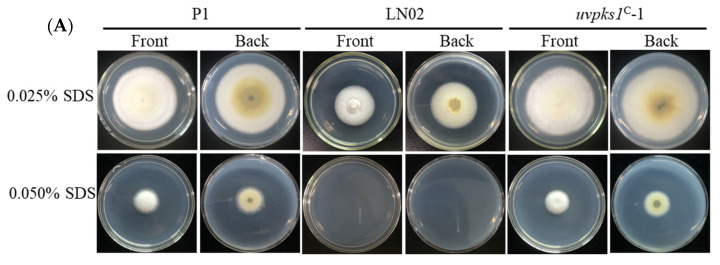

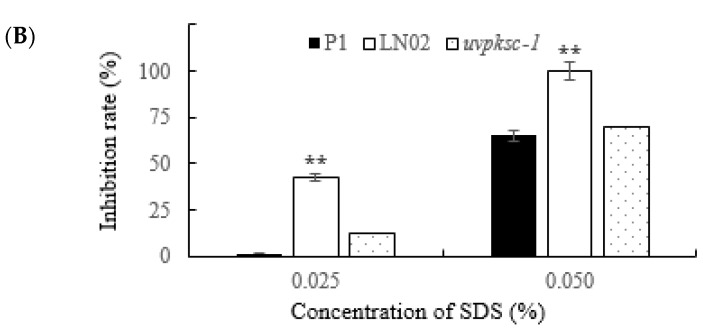
Effects of SDS in medium on growth of fungal strains P1, LN02, and *uvpks1^C^*-1. (**A**) The colonies of fungal strains grew for 21 days after inoculation with SDS at concentrations of 0.025% and 0.050% in PSA medium, respectively. The CK (0.000% SDS) photos of the three strains are the same as those (0.0 M NaCl) in Figure 5A. (**B**) Inhibition of the colony expansion diameters of fungal strains. **, *p* < 0.01.

Secondary metabolites can also increase fungal resistance to cell membrane damage [29]. Sodium dodecyl sulfate (SDS) can perturb membrane integrity. The strains P1, LN02, and *uvpks1^C^*-1 were subjected to cell membrane stress induced by SDS with concentrations of 0.025% and 0.050% in PSA medium, respectively. The growth of strain LN02 was completely inhibited when the SDS was at 0.050%, while those of strains P1 and *uvpks1^C^*-1 were moderately inhibited by the SDS at 0.050% (Figure 7). These results indicate that the activation of *uvpks1* could increase fungal resistance to cell membrane stress induced by SDS.

#### 3.2.6. Activation of *uvpks1* Increased Fungal Resistance to H_2_O_2_-Induced Oxidative Stress

**Figure 8:**
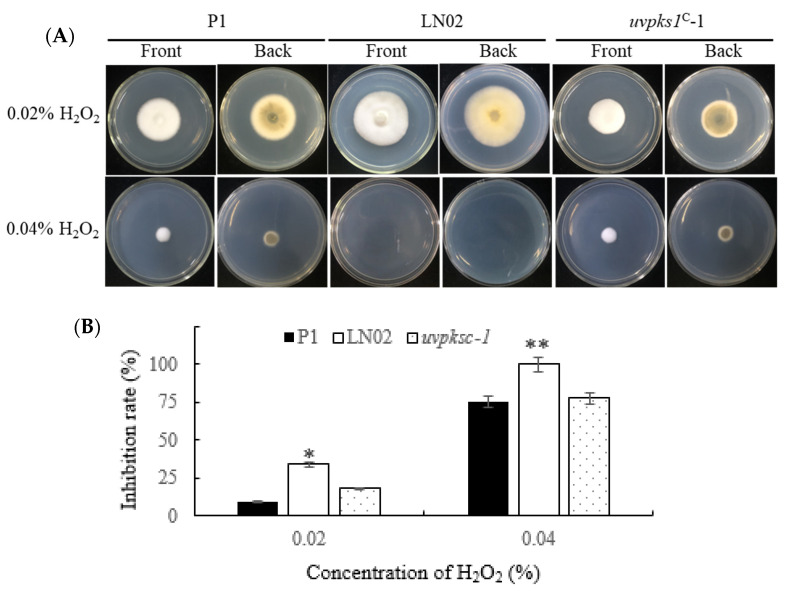
Effects of H_2_O_2_ in medium on growth of fungal strains P1, LN02, and *uvpks1^C^*-1. (**A**) The colonies of fungal strains grew for 21 days after inoculation with H_2_O_2_ at concentrations of 0.02% and 0.04% in PSA medium, respectively. The CK (0.00% H_2_O_2_) photos of the three strains are the same as those (0.0 M NaCl) in Figure 5A. (**B**) Inhibition of the colony expansion diameters of fungal strains. *, *p* < 0.05; **, *p* < 0.01.

Secondary metabolites are usually formed in fungi in response to oxidative stress induced by hydrogen peroxide (H_2_O_2_) [30]. When fungal strains were cultured in the medium containing 0.02–0.04% H_2_O_2_, the mycelial growth and pigment production of strain LN02 were more strongly inhibited than those of strains P1 and *uvpks1^C^*-1 (Figure 8A). Strain LN02 could not grow in the medium containing 0.04% H_2_O_2_ (Figure 8). The LN02 strain showed reduced tolerance to H_2_O_2_ compared with P1, while the complemented strain *uvpks1^C^*-1 showed statistically the same tolerance to stress responses as the wild type. The results indicate that the function of *uvpks1* was restored in the complemented strain *uvpks1^C^*-1.

### 3.3. Activation of uvpks1 Increased Inhibition by the Fungal Extracts of Germination of Rice Seeds

**Figure 9:**
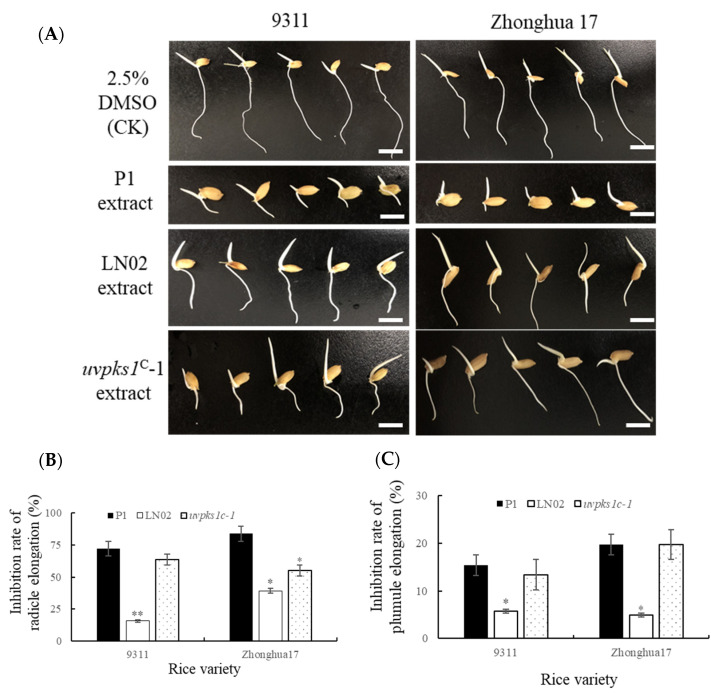
Effects of fungal extracts of the strains on germination of rice seeds. (**A**) Germinated rice seeds were treated with fungal extracts at 10 μg/mL. (**B**) Inhibition of radicle elongation. (**C**) Inhibition of plumule elongation. Scale bar: 0.5 cm in (**A**); *, *p* < 0.05; **, *p* < 0.01.

The EtOAc crude extracts of the fermentation cultures of strains P1, LN02, and *uvpks1^C^*-1 were evaluated for their inhibition of the seed germination of two rice varieties, 9311 and Zhonghua 17. The inhibitions of radical and plumule elongation are shown in Figure 9. Both the radicle (Figure 9B) and plumule (Figure 9C) inhibition rates of the extracts from the strain P1 and *uvpks1^C^*-1 cultures were obviously bigger than those of strain LN02.

Many phytotoxic metabolites produced by plant pathogenic fungi show inhibition of seed germination. They are usually considered pathogenic factors to host plants [31] and exhibit bioherbicidal potential in agriculture [32,33,34]. In our previous report, four main bis-naphtho-γ-pyrones, including ustilaginoidins E, K, and D, and isochaetochromin B2 were identified in the fermentation cultures of strains P1 and *uvpks1^C^* [11]. Ustilaginoidins E, K, D, and B2 were also reported as the main bis-naphtho-γ-pyrones in the fermentation cultures of normal *V. virens*. At the same time, ustilaginoidin E and isochaetochromin B2 were screened to show their inhibitory activity on the radical elongation of rice seeds [17]. It was speculated that the high content of phytotoxic ustilaginoidins in the P1 and *uvpks1^C^*-1 strains may contribute to their inhibition of the germination of rice seeds.

## 4. Discussion

The capacity of fungi to respond to stresses influences their development and virulence, as well as their adaptation to environmental stresses. Fungal secondary metabolites may play crucial roles in controlling morphological differentiation, environmental fitness, and interactions with other organisms [18,35,36,37,38,39,40].

Up to now, three main types of toxic secondary metabolites, including 7 ustiloxins [41,42,43], 27 ustilaginoidins [17,44,45,46], and 21 sorbicillinoids [47,48], have been identified in *V. virens*. These metabolites were assessed to show their cytotoxic, antimicrobial, animal-toxic, and phytotoxic activities [43,47,48,49,50,51,52,53]. Ustilaginoidins are bis-naphtho-γ-pyrone mycotoxins. To the best of our knowledge, the physiological and ecological functions of ustilaginoidins in *V. virens* remain unknown [54].

In our previous study [11], it was found that the polyketide synthase (PKS)-encoding gene *uspks1* for ustilaginoidin biosynthesis in strain LN02 was inactivated due to the deletion of four bases in the promoter sequence of *uvpks1*. Therefore, the albino strain LN02 was considered a white-phenotype mutant with its incapability to synthesize pigments mainly as ustilaginoidins. In addition, four main bis-naphtho-γ-pyrones, including ustilaginoidins E, K, and D, and isochaetochromin B2 were identified in the fermentation cultures of the normal strain P1 [55] and complemented strain *uvpks1^C^* [11]. In this study, the LN02 strain showed a reduced tolerance to stresses compared with the normal strain P1, while the complemented strain *uvpks1^C^*-1 showed statistically the same tolerance to stress responses as the normal strain P1. The activation of *uvpks1* in strain LN02 could increase sporulation, conidia germination, and fungal resistance to a series of stresses, and the inhibition of rice seed germination.

Both ustilaginoidin E and isochaetochromin B2 were screened to show their phytotoxic activity on rice seedlings [17]. These two compounds (ustilaginoidin E and isochaetochromin B2) were also identified as the main ustilaginoidins in the EtOAc extract from the *uvpks1^C^* strain [11], which led to increased sporulation, conidia germination, and inhibition of rice seed germination. It is plausible that ustilaginoidins might be associated with the virulence factors in the interactions of *V. virens* with rice plants. The pathogenic fungi were capable of living in an environment with a wide pH range. While living in symbiosis with plants, the rhizospheric environment is mostly neutral or acidic, which is beneficial for fungal secondary metabolism and infection [56,57,58,59,60,61]. In this study, the growth of the albino strain LN02 was more strongly inhibited with a pH of 5.5 than that of the P1 strain or *uvpks1*^C^- strain 1 (Figure 4). This indicates that the *uvpks1* gene might play a positive role in the pH response for the growth of *V. virens*.

It was speculated that the activation of the ustilaginoidin biosynthesis gene *uvpks1* in the albino strain LN02 led to the production of ustilaginoidins in *V. virens*, which might have contributed to the increase in their resistance to environmental stresses. It also contributed to the increased sporulation, conidia germination, and inhibition of rice seed germination. In addition, at a relatively low temperature (i.e., 23 °C) and pH from 5.5 to 7.5, the activation of the ustilaginoidin biosynthesis gene *uvpks1* could increase the mycelial growth of *V. virens*. Therefore, ustilaginoidins are considered to play important physiological and ecological functions in fungal development, environmental fitness, and interactions with other organisms. However, the albino *V. virens* cannot synthesize ustilaginoidins. This may be the reason why the white RFS rarely occurs in rice fields, which is worthy of further investigation.

## 5. Conclusions

In summary, three strains including the normal strain P1, albino strain LN02, and complemented strain *uvpks1*^C^-1 of *V. virens* were used to investigate the activation of the ustilaginoidin biosynthesis gene *uvpks1* in the albino strain LN02 to influence fungal sporulation, conidia germination, pigment production, stress responses, and the inhibition of rice seed germination. The activation of the ustilaginoidin biosynthesis gene *uvpks1* in the albino strain LN02 increased fungal resistance to NaCl-induced osmotic stress, Congo-red-induced cell wall stress, SDS-induced cell membrane stress, and H_2_O_2_-induced oxidative stress. It also increased sporulation, conidia germination, and the inhibition of rice seed germination. In addition, at a relatively low temperature (i.e., 23 °C) and pH from 5.5 to 7.5, the activation of the ustilaginoidin biosynthesis gene *uvpks1* was able to increase the mycelial growth of *V. virens*. The findings indicate that *uvpks1* could influence the development, pigment production, stress responses, and inhibition of rice seed germination by controlling ustilaginoidin biosynthesis in *V. virens*. The albino strain LN02 of *V. virens* should be an ideal fungal material to study the biological functions (i.e., protecting *V. virens* from various stresses, and interactions between *V. virens* and rice plants) of ustilaginoidins. However, the action mechanisms of ustilaginoidins corresponding to their biological functions, such as the interactions between *V. virens* and its host rice plant, need further investigation. Studies about whether the complemented strain *uvpks1^C^* of the albino strain LN02 can produce normal false smut balls, or whether the deletion strain Δ*uvpks1* from the normal strain P1 can produce white false smut balls, on rice in fields are in progress.

## Data Availability

Data are contained within the article and Supplementary Materials.

## Supplementary Materials

The following are available online at https://www.mdpi.com/article/10.3390/jof10010031/s1: Establishment of the complemented strain *uvpks1^C^*; Table S1: The primers used in this study.
