# Supplementary material for: Activation of Ustilaginoidin Biosynthesis Gene uvpks1 in Villosiclava virens Albino Strain LN02 Influences Development, Stress Responses, and Inhibition of Rice Seed Germination
Source: J Fungi (Basel). 2023 Dec 31;10(1):31. doi: 10.3390/jof10010031 (PMC10817433; doi:10.3390/jof10010031)
Supplement: Supplementary file 1 [file jof-10-00031-s001.zip › jof-2752459-supplementary.pdf]

Supplementary Materials:

Activation of Ustilaginoidin Biosynthesis Gene *uvpks1* in *Villosiclava virens* Albino Strain LN02 Influences Development, Stress Responses, and Inhibition of Rice Seed Germination

Mengyao Xue, Xuwen Hou, Gan Gu, Jie Dong, Yonglin Yang, Xiaoqian Pan, Xuan Zhang, Dan Xu, Daowan Lai, and Ligang Zhou *

Department of Plant Pathology, College of Plant Protection, China Agricultural University, Beijing 100193, China
* Correspondence: lgzhou@cau.edu.cn

Establishment of the complemented strain *uvpks1*^{C}

The open reading frame (ORF) sequence of *uvpks1* containing a normal native promoter in the strain P1 of *Villosiclava virens* was cloned into the pCBHT binary vector. The vector was introduced into the protoplasts of strain LN02 of *V. virens*. The complemented strains *uvpks1*^{C}-1, *uvpks1*^{C}-2, and *uvpks1*^{C}-3 were detected to restore the ability to synthesize ustilaginoidins (Xue M, et al. *Int. J. Mol. Sci.* **2023**, *24*, 15196). The primers used were listed in Table S1. All strains were grown in the incubator at 28 °C. The pCBHT vector was kindly provided by Prof. Jin-Rong Xu from Department of Botany and Plant Pathology, Purdue University, West Lafayette, IN, USA.

Table S1. The primers used in this study.

Primer	Oligonucleotide Sequence (5′-3′)	Description
*Uvpks1*F-2	ATGGCGAACGTGTTCCAAATTG	The primers were used to amplify the sequences of *uvpks1*.
*Uvpks1*R-4	GGCGTTGATCTCGCGGTAGTC	
*Uvpks1*F-5	TCGACTCGAGGTTCTTCAGCA	
*Uvpks1*R-5	CAGGGTCGACCTAAGCATGTTG	
*Uvpks1*F-1	ATGAAGAGCGCCTCGGACAT	
Uvpks1-R	TTAGATACCCGCGCCGGTAGA	
Uvpks1-pro-P1-F	ACGACCACGACCACGACGA	The primers were used to amplify the sequences of the promoter of *uvpks1* in P1 strain.
Uvpks1-pro-P1-R	TACGGCGAAAGATGTCGAAACGAG	
Uvpks1-pro-LN02-F	ACGACCACGACCACGACGA	The primers were used to amplify the sequences of the promoter of *uvpks1* in LN02 strain.
Uvpks1-pro-LN02-R	TACGGCGAAAGATGTCGAAACGAG	
Uvpks1-pCBHT-F	aatgatggatccccgggtaccccACGACCACGACCACGACGA	The primers were used to construct the complementary

Uvpks1-pCBHT-R	agaaatcgcaacctcgaattcTTAGATACCCGCG CCGGTAGA	vectors.
Uvpks1-GFP-P1-F	gcccttgctcaccatggatccACGACCACGACCA CGACGA	The primers were used to construct the 1305-GFP vectors for transient expression assay of P1 strain.
Uvpks1-GFP-P1-R	aagtccggagctagctctagaTACGGCGAAAGA TGTCGAAACGAG	
Uvpks1-GFP-LN02-F	gcccttgctcaccatggatccACGACCACGACCA CGACGA	The primers were used to construct the 1305-GFP vectors for transient expression assay of LN02 strain.
Uvpks1-GFP-LN02-R	aagtccggagctagctctagaTACGGCGAAAGA TGTCGAAACGAG	
